# Drug-Coated Balloon in Primary Percutaneous Coronary Intervention

**DOI:** 10.1155/2023/5210808

**Published:** 2023-06-26

**Authors:** Hui Zhao, Runran Miao, Fei Lin, Guoan Zhao

**Affiliations:** First Affiliated Hospital of Xinxiang Medical University, Xinxiang, Henan 453100, China

## Abstract

According to the latest coronary interventional guidelines, a drug-eluting stent is the recommended reperfusion therapy in primary percutaneous coronary intervention (pPCI). However, deficiencies and defects, such as in-stent restenosis (ISR), incomplete stent apposition, stent thrombosis, reinfarction after stent implantation, long-term dual antiplatelet drug use, and adverse reactions of metal implants, plague clinicians and patients. Drug-coated balloon (DCB), which delivers antiproliferative agents into the vessel wall without stent implantation and leaves no implants behind after the procedure, is a novel option for percutaneous coronary intervention and has proven to be a promising strategy in cases of ISR, small vessel coronary artery disease, and bifurcation lesions. However, most of the available experience has been gained in elective percutaneous coronary intervention, and experience in pPCI is lacking. The current evidence for the use of DCB-only in pPCI was discussed and analyzed in this review.

## 1. Introduction

Although advances have been made in emergency treatment, acute myocardial infarction (AMI) remains the leading cause of death worldwide [1]. Primary percutaneous coronary intervention (pPCI) is the best treatment modality [2]. With the development of devices and advances in surgical techniques, drug-eluting stents (DESs) have become the preferred option for interventional procedures and were proven to be an effective and safe treatment for acute coronary syndrome (ACS) in pPCI [2]. DES was recommended as a Class I indication by guidelines for the management of ST-elevation myocardial infarction (STEMI) published by the European Society of Cardiology (ESC) in 2017 [3, 4]. Until today, pPCI implantation of DES has been recommended because the technique shows excellent immediate revascularization results as well as good medium- to long-term clinical outcomes [5].

With the use of DESs and the development of long-term observational studies, many shortcomings of DES have come to the fore. In this context, the drug-coated balloon (DCB), a semicompliant balloon with drug coated around the outside, may offer an attractive treatment modality as an emerging percutaneous coronary interventional device. The main disadvantage of the stent strategy comes from the metal residue. The DCB-only strategy without stenting is increasingly accepted by patients. However, evidence of effectiveness and safety is still lacking [6, 7]. This review discussed the current studies using DCB-only in pPCI and provided a preliminary analysis of the results.

## 2. Challenges of DES

To prevent stent thrombosis in the early or late stages of implantation, we need to use long-term dual antiplatelet therapy, which increases the risk of bleeding [8, 9]. Some bleeding events can lead to death, especially in older or low glomerular filtration rate populations with dual antiplatelet therapy [10]. Intrastent restenosis (ISR) causes recurrence of ACS and makes retreatment of coronary arteries more difficult, which is a troublesome problem with stents [11]. Highly calcified lesions increase the risk of incomplete stent apposition (ISA). Several devices and equipment have been developed to improve the outcome of stents. For example, rotational atherectomy and intravascular lithotripsy have decreased the risk of ISA [12–14], and intravascular ultrasound (IVUS) and optical coherence tomography (OCT) have optimized stent implantation results [15–17]. However, the risks associated with stents cannot be ignored, especially in pPCI. For example, ISA is more frequent in DESs implanted during pPCI than in DESs implanted for stable/unstable angina [18].

## 3. The Overview of DCB Technology

DCB allows delivery of antiproliferative topical agents directly into the coronary wall after a single balloon inflation [19]. DCB-only strategy fulfills the concept of “implant-free,” which prevents any potential problem caused by stents. A shorter duration of antiplatelet therapy was needed, which reduces the risk of bleeding [20, 21] and avoids the increased risk of thrombosis associated with implants such as stents [22].

Paclitaxel and rapamycin are attached to the exterior of the balloon as inhibitors of vascular endothelial proliferation and have been shown to be successful in preventing vascular endothelial hyperplasia [23]. Most DCB surfaces use paclitaxel due to its high lipophilic characteristics that allow passive absorption through the cell membranes and a persistent effect inside the target vessel wall. However, that dominance is now being challenged. Some studies suggest possible risks of paclitaxel, such as the possibility of increased long-term mortality [24, 25]. Sirolimus is being coated on the balloon surface as a new inhibitor and is being introduced into the interventional field, and some evidence has been obtained in clinical trials [26–28]. However, only 3 sirolimus DCBs were approved for clinical use, and clinical data for sirolimus-coated balloons are still scarce compared to paclitaxel-coated balloons.

## 4. DCB-Only Strategy in pPCI

When DCB was the first used to treat ISR, several randomized trials confirmed its efficacy and safety profile in small-vessel disease and high bleeding risk [29]. Subsequently, several emerging indications for DCBs were identified, such as bifurcation lesions, large-vessel diseases, diabetes mellitus, and ACS [30–32]. This strategy using DCB-only without stenting is increasingly accepted by patients and has demonstrated its effectiveness and safety. The main disadvantage of stents strategy comes from the metal residue, whereas the DCB-only strategy has the advantage of providing antiproliferative agents to the endothelium without leaving any implants.

There are studies on the use of DCB-only strategies in pPCI, including prospective studies or retrospective analyses evaluating the safety and efficacy of DCB-only strategies in pPCI and whether they are inferior to DESs (Table 1).

Vos et al. may have conducted the first study to evaluate the safety and feasibility of using only DCB in STEMI patients undergoing pPCI [33]. In this prospective single-arm study, a total of 100 STEMI patients underwent pPCI, 59 of whom were treated with DCB-only, and 41 required additional stenting because they developed C-to-F coronary dissection or residual stenosis >50%. It was the first study using a DCB-only angioplasty strategy in the setting of pPCI and showed good one-year clinical outcomes. Of the 98 who completed the 1-year follow-up, 5 had MACE, 2 had cardiac death, and 3 received target lesion revascularization (TLR).

Ho et al. also conducted a single-arm retrospective study of the clinical feasibility of using DCB-only in STEMI patients undergoing pPCI [34]. All 89 STEMI patients included received DCB during pPCI, and 4% of them received compensatory stenting. At the 30-day follow-up, there were four deaths. In this study, using DCB-only pPCI was feasible. It was necessary for better contact such as aspiration of visible thrombus prior to DCB angioplasty, adequate predilation, and extended balloon inflation.

To compare the safety and differences in late vascular lumen conditions in STEMI patients treated with DCB or DES, Gobić et al. conducted a prospective controlled study [35]. 75 patients with STEMI were randomized to the DES group (*n* = 37) and the DCB group (*n* = 38) and received 6-month follow-up. In this study, DCB-only strategy was safe and feasible and showed good clinical and angiographic outcomes in a 6-month follow-up period. MACE occurred in 5.4% of patients in the DES group and none in the DCB group (*P*=0.29). LLL was 0.10 ± 0.19 mm in the DES group and −0.09 ± 0.09 mm in the DCB group (*P* < 0.05).

Zhang et al. conducted a single-center clinical trial to compare the safety and clinical outcomes of the DCB strategy with the DES strategy in pPCI in patients with AMI [36]. 380 patients received a 3-month follow-up. The incidence of MACE during hospitalization was similar in both groups (DCB group 3.3% (6/180) and DES group 1.0% (2/200), *P*=0.15) which was mostly associated with delayed coronary artery dissection, and 1 death occurred in each group. In this study, the safety and efficacy of the DCB strategy were similar to DES. No MACE occurred in either group within 3 months after discharge, while the difference in the incidence of bleeding events was not statistically significant (*P*=0.91). The incidence of coronary artery dissection was significantly higher in the DCB group than in the DES group (8.3% (15/180) and 3.0% (6/200), *P*=0.02), but most of them were type B or A dissections and did not need special treatment.

Hao et al. conducted a randomized controlled clinical trial to study late lumen loss after DCB treatment in pPCI among STEMI patients [37]. 80 patients, randomized to the DCB-treated group (*n* = 38) and the DES group (*n* = 42), were reviewed by coronary angiography for late lumen loss (LLL) in both groups at 1 year postoperatively, and their incidence of MACE at 1 month, 6 months, and 1 year postoperatively was recorded. The results showed that DCB without stenting in pPCI for STEMI was safe and effective during the one-year follow-up period. The DCB group had less target lesion LLL after 1 year compared to the DES group (−0.12 ± 0.46 mm vs 0.14 ± 0.37 mm, *P* < 0.05), while the incidence of MACE was not significantly different (11% (4/38) in the DCB group vs 12% (5/42) in the DES group).

Niehe et al. evaluated the efficacy of DCB and DES regimens after 2 years in the DCB versus DES revascularization in STEMI study [38]. 109 patients (91%) had complete clinical follow-up at 2 years. In this study, the DCB and DES groups had the same 2-year clinical outcomes. MACE occurred in 3 patients (5.4%) in the DCB group and 1 patient (1.9%) in the DES group (OR, 2.86; 95% confidence interval, 0.30–27.53; *P*=0.34).

Duan et al. compared the safety and clinical outcomes of DCB versus DES strategies in pPCI in AMI patients through a single-center retrospective study [39]. The 126 patients selected from 213 STEMI patients by inclusion criteria and propensity score matching (PMS) were divided into DCB and DES groups and received 1-year follow-up. Before PMS, the rate of major adverse cardiovascular events (MACE) occurrence was almost the same (16.28% (21/129) VS. 14.29% (12/84) OR 1.17, 95% CI: 0.54 to 2.52, *P*=0.69). After PMS, more MACEs occurred in the DES group than in the DCB group (24.2% (15/62) VS 9.7% (6/62) OR: 2.50, 95% CI 1.04 to 6.02, *P*=0.04). The results showed that DCB was a possible strategy for pPCI in STEMI patients.

## 5. Advantages and Disadvantages of the DCB-Only Strategy in pPCI

**Figure 1:**
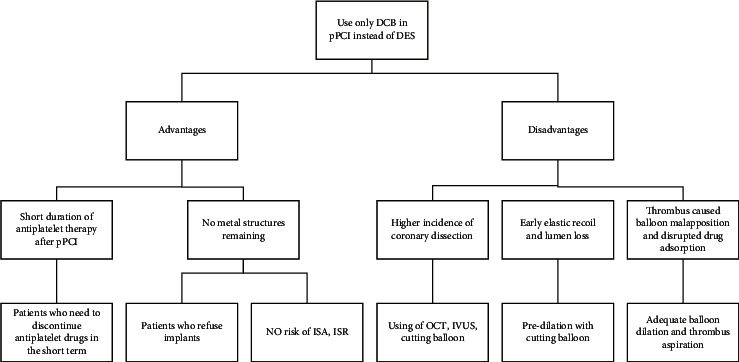
Advantages and disadvantages of using DCB-only in pPCI instead of DES. DCB, drug-coated balloon; pPCI, primary percutaneous coronary intervention; DES, drug-eluting stent; ISR, intrastent restenosis; ISA, incomplete stent apposition; OCT, optical coherence tomography; IVUS, intravascular ultrasound.

Considering the above studies, DCB-only may be a safe and effective option for pPCI. DCB-only is a possible treatment option for pPCI in patients with contraindications to DES or who are not suitable for immediate DES implantation. However, these studies also reflect the shortcomings of the DCB strategy and provide some ideas for improving them. They are summarized in Figure 1. Residual thrombus in the coronary artery during pPCI can prevent adequate drug release into the vessel wall. Aspirating a visible thrombus prior to DCB angioplasty, performing adequate predilation, and prolonging balloon filling time were suggested to achieve better contact. The incidence of coronary artery dissection after DCB was higher than that of DES, which may lead to remedial stent implantation for even more in-hospital MI [33]. Experienced physicians, proper pretreatment, and the use of endovascular luminal aids can improve outcomes and reduce complications. For example, IVUS and OCT can provide proper protocol guidance to decrease the incidence of dissection or identify hidden dissections for remedial treatment [40]. Cutting the balloon can reduce postoperative elastic retraction of the vessel and lumen loss due to a lack of bracing [41, 42]. Compared to conventional balloons, predilation with cutting balloon was proven to be able to reduce the incidence of severe dissections (types E and F) [43, 44].

## 6. Conclusions

Although DES remains the standard reperfusion strategy in the cases of most AMI lesions, initial evidence, clinical practice, and theoretical evidence point towards DCB-only which appears to be a promising strategy in pPCI. While the clinical data supporting the use of DCB-only in pPCI are limited, research currently exists showing that the use of DCB can reduce the total number and length of stents and even avoid implantation in suitable patients, with undiminished efficacy, which represents a huge allure for patients, especially young people or patients who are reluctant or unable to undergo implantation for any reason.

## Figures and Tables

**Table 1:** Clinical trials of DCBs for the treatment of primary percutaneous coronary intervention.

Researchers (year) (reference)	Group size (*n*)	Follow-up (month)	Endpoint	Outcome (DCB vs. DES)	Conclusion
Vos et al. 2014 [33]	DCB: 100	12	MACE, TLR	5 MACE, 2 death, 3 received TLR	DCB without stenting showed good one-year clinical outcomes
Ho et al. 2015 [34]	DCB: 89	1	Compensatory stenting, death	4% patients received compensatory stenting, 4 patients dead in 1 month	DCB-only is feasible
Gobić et al. 2017 [35]	DCB: 38/DES: 37	6	MACE, LLL	MACE: 0.0% (0/38) VS. 5.4% (2/37), *P*=0.29 LLL: −0.09 ± 0.09 mm VS. 0.10 ± 0.19 mm	DCB-only strategy was safe, feasible and effective
Zhang et al. 2020 [36]	DCB: 180/DES: 200	3	MACE, coronary artery dissection	MACE: 3.3% (6/180) VS. 1.0% (2/200), *P*=0.15 Coronary artery dissection: 8.3% (15/180) VS. 3.0% (6/200), *P*=0.02	DCB-only had the same safety and efficacy as stents
Hao et al. 2021 [37]	DCB: 38/DES: 42	12	MACE, LLL	MACE: 11% (4/38) vs. 12% (5/42) LLL: −0.12 ± 0.46 mm vs 0.14 ± 0.37 mm, *P* < 0.05	DCB without stenting is safe and effective
Niehe et al. 2022 [38]	DCB: 56/DES: 53	24	MACE	5.4% (3/56) VS. 1.9% (1/53), *P*=0.34	The DCB group had same 2-yearclinical outcomes to DES group
Duan et al. 2022 [39]	DCB: 84/DES: 129	12	MACE	Before PMS: 14.29% (12/84) VS. 16.28% (21/129), *P*=0.69 After PMS: 9.7% (6/62) vs. 24.2% (15/62), *P*=0.04	DCB-only was a possible strategy for pPCI

DCB, drug-coated balloon; DES, drug-eluting stent; MACE, major adverse cardiovascular events; PMS, propensity matching score; LLL, late lumen loss; TLR, target lesion revascularization; BMS, bare metal stent; pPCI, primary percutaneous coronary intervention.

## Data Availability

No new data are applicable to this study.

## References

[1] Liu W., Shen J., Li Y. (2021). Pyroptosis inhibition improves the symptom of acute myocardial infarction. *Cell Death & Disease*.

[2] Sousa-Uva M., Neumann F.-J., Ahlsson A. (2018). ESC/EACTS Guidelines on myocardial revascularization. *European Journal of Cardio-Thoracic Surgery: Official Journal of the European Association for Cardio-Thoracic Surgery*.

[3] Ozaki Y., Hara H., Onuma Y. (2022). CVIT expert consensus document on primary percutaneous coronary intervention (PCI) for acute myocardial infarction (AMI) update 2022. *Cardiovascular intervention and therapeutics*.

[4] Ibanez B., James S., Agewall S. (2017). ESC Guidelines for the management of acute myocardial infarction in patients presenting with ST-segment elevation: the Task Force for the management of acute myocardial infarction in patients presenting with ST-segment elevation of the European Society of Cardiology (ESC). *European Heart Journal*.

[5] Katz G., Harchandani B., Shah B. (2015). Drug-eluting stents: the past, present, and future. *Current Atherosclerosis Reports*.

[6] Geng B., Liu Z., Feng G., Jiang J. (2021). Drug-coated balloon versus drug-eluting stent in acute myocardial infarction: a protocol for systematic review and meta-analysis. *Medicine*.

[7] Li Q. Y., Chang M. Y., Wang X. Y. (2022). Efficacy and safety of drug-coated balloon in the treatment of acute myocardial infarction: a meta-analysis of randomized controlled trials. *Scientific Reports*.

[8] Fukuizumi I., Tokita Y., Shiomura R. (2023). Angioscopic findings 1 year after percutaneous coronary intervention for chronic total occlusion. *Journal of Cardiology*.

[9] Bittl J. A., Baber U., Bradley S. M., Wijeysundera D. N. (2016). Duration of dual antiplatelet therapy: a systematic review for the 2016 ACC/AHA guideline focused update on duration of dual antiplatelet therapy in patients with coronary artery disease: a report of the American college of Cardiology/American heart association task force on clinical practice guidelines. *Circulation*.

[10] Heindl B., Clarkson S., Parcha V. (2022). Risk of postdischarge bleeding from dual antiplatelet therapy after percutaneous coronary intervention among US black and white adults. *Journal of the American Heart Association*.

[11] Alraies M. C., Darmoch F., Tummala R., Waksman R. (2017). Diagnosis and management challenges of in-stent restenosis in coronary arteries. *World Journal of Cardiology*.

[12] Mastrangelo A., Monizzi G., Galli S. (2022). Intravascular lithotripsy in calcified coronary lesions: a single-center experience in “Real-World” patients. *Frontiers in cardiovascular medicine*.

[13] Iwańczyk S., Włodarczak A., Hiczkiewicz J. (2021). Feasibility of intravascular lithotripsy for calcific coronary lesions: a multi-institutional experience. *Catheterization and Cardiovascular Interventions*.

[14] Sakakura K., Ito Y., Shibata Y. (2021). Clinical expert consensus document on rotational atherectomy from the Japanese association of cardiovascular intervention and therapeutics. *Cardiovascular intervention and therapeutics*.

[15] Ge Z., Gao X. F., Kan J. (2021). Comparison of one-month versus twelve-month dual antiplatelet therapy after implantation of drug-eluting stents guided by either intravascular ultrasound or angiography in patients with acute coronary syndrome: rationale and design of prospective, multicenter, randomized, controlled IVUS-ACS and ULTIMATE-DAPT trial. *American Heart Journal*.

[16] Hu M. J., Tan J. S., Yin L. (2022). Clinical outcomes following hemodynamic parameter or intravascular imaging-guided percutaneous coronary intervention in the era of drug-eluting stents: an updated systematic review and bayesian network meta-analysis of 28 randomized trials and 11,860 patients. *Frontiers in cardiovascular medicine*.

[17] Gupta A., Chhikara S., Singh N., Prasad K. (2021). Optical coherence tomography-guided management of underexpanded stent in calcified coronary lesion. *BMJ Case Reports*.

[18] Gonzalo N., Barlis P., Serruys P. W. (2009). Incomplete stent apposition and delayed tissue coverage are more frequent in drug-eluting stents implanted during primary percutaneous coronary intervention for ST-segment elevation myocardial infarction than in drug-eluting stents implanted for stable/unstable angina: insights from optical coherence tomography. *JACC: Cardiovascular Interventions*.

[19] Scheller B., Speck U., Abramjuk C., Bernhardt U., Böhm M., Nickenig G. (2004). Paclitaxel balloon coating, a novel method for prevention and therapy of restenosis. *Circulation*.

[20] Valgimigli M., Bueno H., Byrne R. A. (2017). ESC focused update on dual antiplatelet therapy in coronary artery disease developed in collaboration with EACTS: the Task Force for dual antiplatelet therapy in coronary artery disease of the European Society of Cardiology (ESC) and of the European Association for Cardio-Thoracic Surgery (EACTS). *European Heart Journal*.

[21] Sinnaeve P. R., Adriaenssens T. (2021). Dual antiplatelet therapy de-escalation strategies. *The American Journal of Cardiology*.

[22] Nestelberger T., Kaiser C., Jeger R. (2020). Drug-coated balloons in cardiovascular disease: benefits, challenges, and clinical applications. *Expert Opinion on Drug Delivery*.

[23] Zhang D.-M., Chen S. (2020). In-stent restenosis and a drug-coated balloon: insights from a clinical therapeutic strategy on coronary artery diseases. *Cardiology Research and Practice*.

[24] Katsanos K., Spiliopoulos S., Kitrou P., Krokidis M., Karnabatidis D. (2018). Risk of death following application of paclitaxel-coated balloons and stents in the femoropopliteal artery of the leg: a systematic review and meta-analysis of randomized controlled trials. *Journal of the American Heart Association*.

[25] Sato Y., Kuntz S. H., Surve D. (2019). What are the pathological concerns and limitations of current drug-coated balloon Technology?. *Heart International*.

[26] Ahmad W. A. W., Nuruddin A. A., Abdul Kader M. A. S. K. (2022). Treatment of coronary de novo lesions by a sirolimus- or paclitaxel-coated balloon. *JACC: Cardiovascular Interventions*.

[27] Verheye S., Vrolix M., Kumsars I. (2017). The SABRE trial (sirolimus angioplasty balloon for coronary in-stent restenosis): angiographic results and 1-year clinical outcomes. *JACC: Cardiovascular Interventions*.

[28] Ali R. M., Abdul Kader M. A. S. K., Wan Ahmad W. A. (2019). Treatment of coronary drug-eluting stent restenosis by a sirolimus- or paclitaxel-coated balloon. *JACC: Cardiovascular Interventions*.

[29] Ang H., Koppara T. R., Cassese S., Ng J., Joner M., Foin N. (2020). Drug-coated balloons: technical and clinical progress. *Vascular Medicine*.

[30] Jeger R. V., Eccleshall S., Wan Ahmad W. A. (2020). Drug-coated balloons for coronary artery disease: third report of the international DCB consensus group. *JACC: Cardiovascular Interventions*.

[31] Buono A., Maffeo D., Pellicano M., de Blasio G., Tespili M., Ielasi A. (2021). Back to the future: the role of DCB for the treatment of coronary bifurcation. *Reviews in Cardiovascular Medicine*.

[32] Cortese B., Sanchez-Jimenez E. (2021). Back to the future: DCB use instead of DES for the treatment of complex, native coronary artery disease.

[33] Vos N. S., Dirksen M. T., Vink M. A. (2014). Safety and feasibility of a PAclitaxel-eluting balloon angioplasty in Primary Percutaneous coronary intervention in Amsterdam (PAPPA): one-year clinical outcome of a pilot study. *EuroIntervention*.

[34] Ho H. H., Tan J., Ooi Y. W. (2015). Preliminary experience with drug-coated balloon angioplasty in primary percutaneous coronary intervention. *World Journal of Cardiology*.

[35] Gobić D., Tomulić V., Lulić D. (2017). Drug-coated balloon versus drug-eluting stent in primary percutaneous coronary intervention: a feasibility study. *The American Journal of the Medical Sciences*.

[36] Zhang D. P., Wang L. F., Liu Y. (2020). Efficacy comparison of primary percutaneous coronary intervention by drug-coated balloon angioplasty or drug-eluting stenting in acute myocardial infarction patients with de novo coronary lesions. *Zhonghua Xinxueguanbing Zazhi*.

[37] Hao X., Huang D., Wang Z., Zhang J., Liu H., Lu Y. (2021). Correction to: study on the safety and effectiveness of drug-coated balloons in patients with acute myocardial infarction. *Journal of Cardiothoracic Surgery*.

[38] Niehe S. R., Vos N. S., van der Schaaf R. J. (2022). Two-year clinical outcomes of the REVELATION study: sustained safety and feasibility of paclitaxel-coated balloon angioplasty versus drug-eluting stent in acute myocardial infarction. *Journal of Invasive Cardiology*.

[39] Duan Y., Wang Y., Zhang M. (2022). Computational pressure-fluid dynamics applied to index of microcirculatory resistance, predicting the prognosis of drug-coated balloons compared with drug-eluting stents in STEMI patients. *Frontiers in Physiology*.

[40] Maehara A., Matsumura M., Ali Z. A., Mintz G. S., Stone G. W. (2017). IVUS-guided versus OCT-guided coronary stent implantation: a critical appraisal. *JACC. Cardiovascular imaging*.

[41] Kawaguchi K., Kondo T., Shumiya T. (2002). Reduction of early elastic recoil by cutting balloon angioplasty as compared to conventional balloon angioplasty. *Journal of Invasive Cardiology*.

[42] Montorsi P., Galli S., Fabbiocchi F., Trabattoni D., Ravagnani P. M., Bartorelli A. L. (2004). Randomized trial of conventional balloon angioplasty versus cutting balloon for in-stent restenosis. Acute and 24-hour angiographic and intravascular ultrasound changes and long-term follow-up. *Italian Heart Journal: Official Journal of the Italian Federation of Cardiology*.

[43] Han B., Aboud M., Nahir M., Noem F., Hasin Y. (2005). Cutting balloons versus conventional long balloons for PCI of long coronary lesions. *International Journal of Cardiovascular Interventions*.

[44] Muramatsu T., Tsukahara R., Ho M. (2002). Effectiveness of cutting balloon angioplasty for small vessels less than 3.0 mm in diameter. *Journal of Interventional Cardiology*.

